# Supplementary material for: Dramatic uneven urbanization of large cities throughout the world in recent decades
Source: Nat Commun. 2020 Oct 23;11:5366. doi: 10.1038/s41467-020-19158-1 (PMC7584620; doi:10.1038/s41467-020-19158-1)
Supplement: Supplementary file 1 — Supplementary Information [file 41467_2020_19158_MOESM1_ESM.pdf]

Supplementary Information

Dramatic uneven urbanization of large cities throughout the world in recent decades

Sun et al.

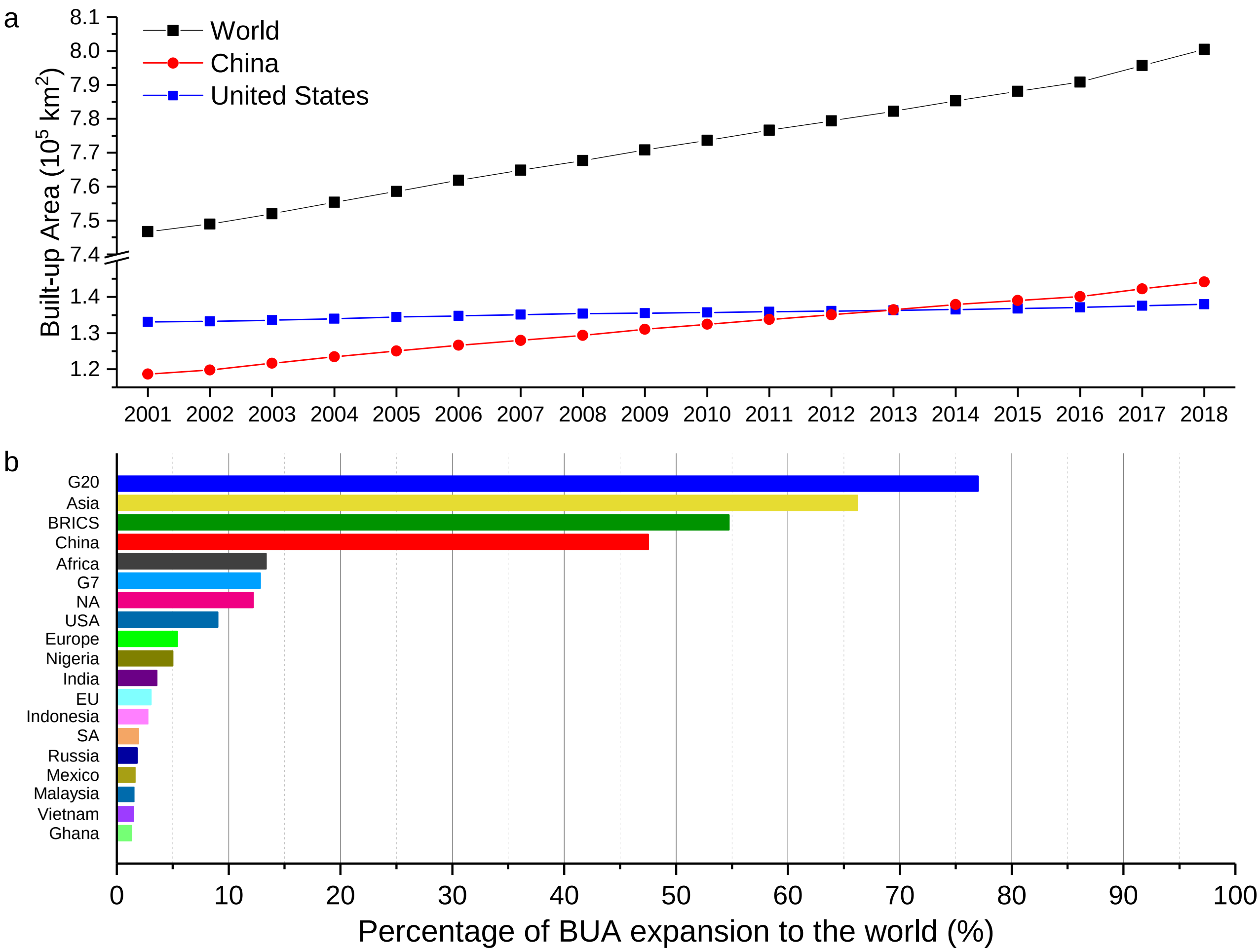

Supplementary Figure 1 | Total built-up area (BUA) changes from 2001 to 2018. **a**. Changes of the total BUA in the world (black line), the USA (blue line), and China (red line) from 2001 to 2018. **b**. Percentages of the total BUA expansion of the world for different continents, international organizations and the 10 countries with largest BUA expansion during the period 2001-2018. They include the 20 members of the Group of Twenty (G20): Argentina, Australia, Brazil, Canada, China, the European Union (EU), France, Germany, India, Indonesia, Italy, Japan, Mexico, Russia, Saudi Arabia, South Africa, South Korea, Turkey, the United Kingdom, and the United States. The Group of Seven (G7) includes 7 advanced countries: Canada, France, Germany, Italy, Japan, the United Kingdom, and the United States. BRICS is the acronym used for the association of 5 major emerging national economies: Brazil, Russia, India, China and South Africa. NA is North America. SA is South America.

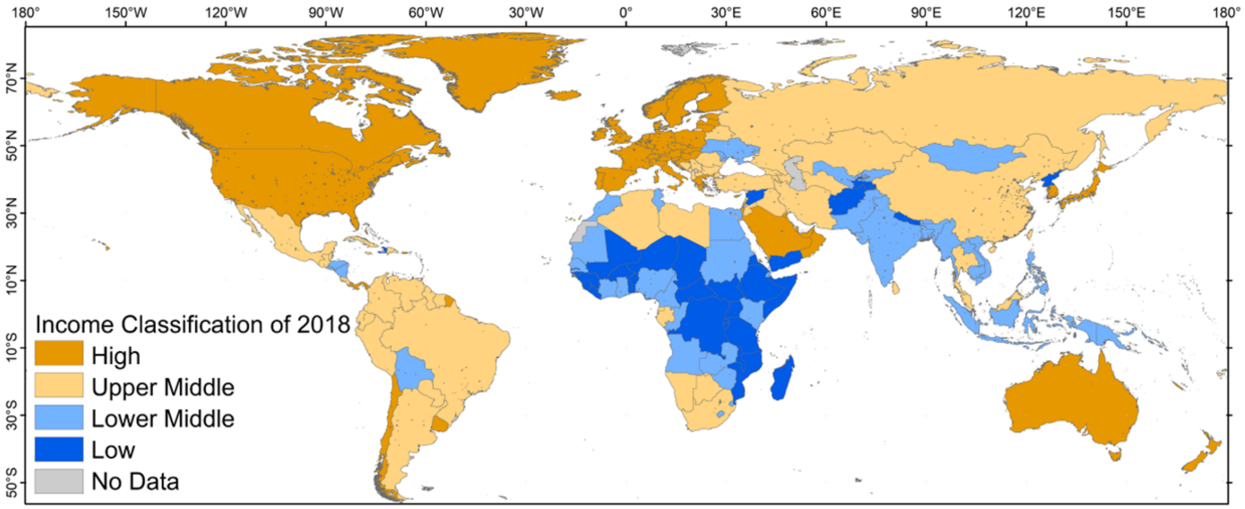

Supplementary Figure 2 | World map of four economic levels in 2018. The map data were classified using the World Bank estimates of 2018 gross national income (GNI) per capita (current US dollars). The World Bank classifies economies as low-income (less than $1,025), lower-middle-income ($1,026–$3,995), upper-middle-income ($3,996–$12,375) or high-income (more than $12,375) as per GNI per capita in 2018.

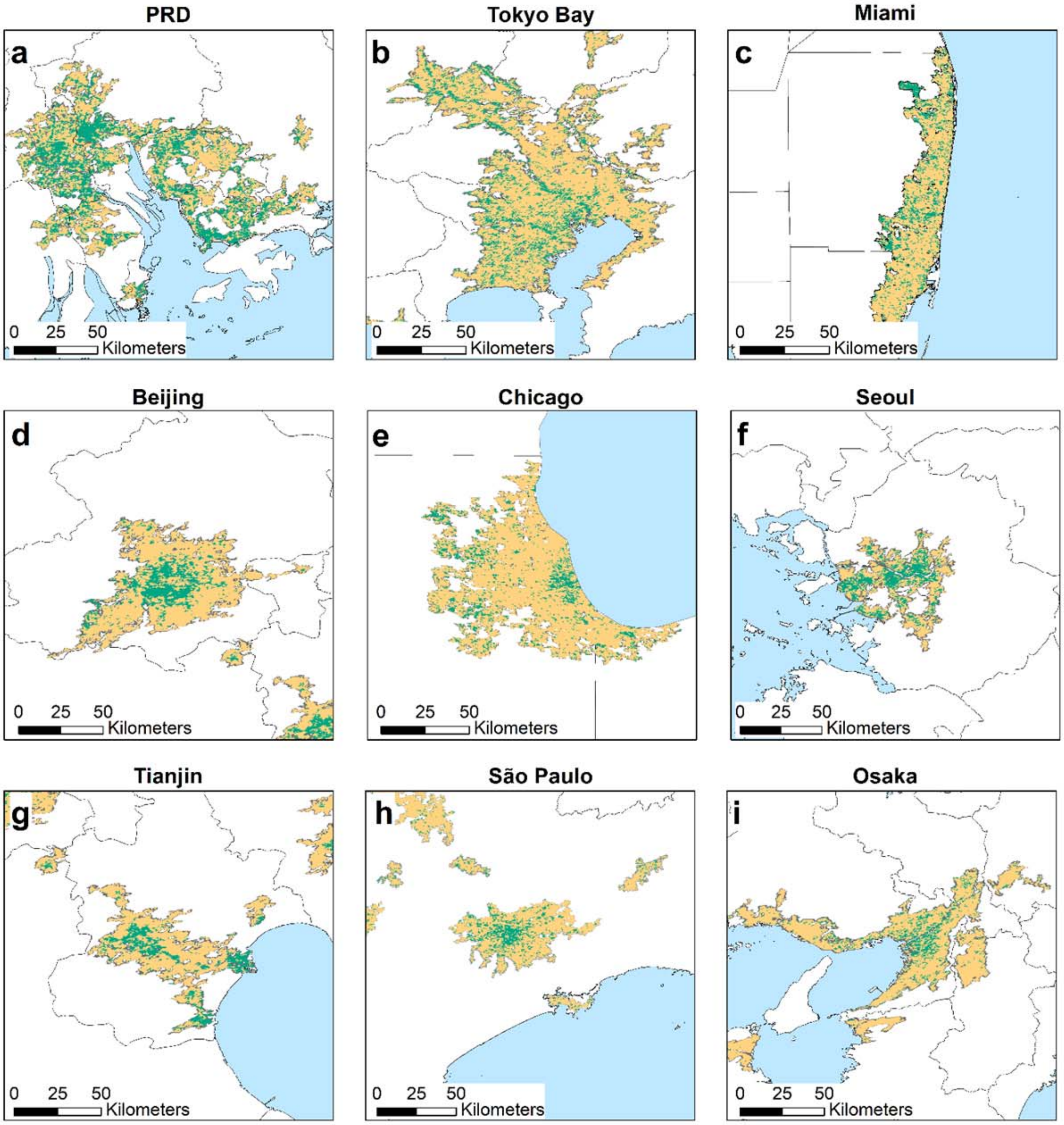

Supplementary Figure 3 | Distribution of 9 large cities with largest area of built-up area (BUA) greening. Distribution of urban pixels (light yellow) with significant ($P < 0.05$) EVI_{max} increment (green) of the nine large cities. All of the scale bars are the same from **a** to **i**. PRD: Pearl River Delta. The black lines are the boundaries of the regions from Level 1 file in the Database of Global Administrative Areas (GADM) (see the details in Methods).

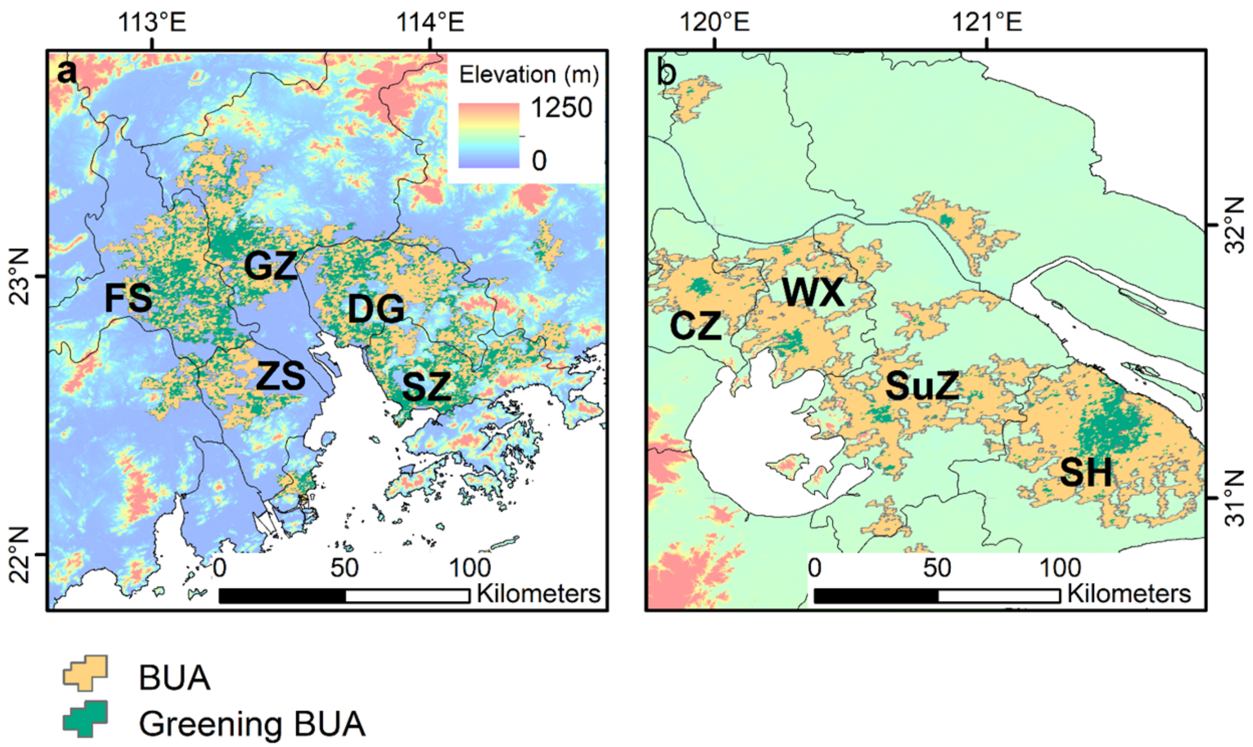

Supplementary Figure 4 | Distribution of the greening built-up area (BUA) and elevation of Pearl River Delta (PRD) and Yangtze River Delta (YRD) urban agglomerations. Urban areas with significant EVI_{max} increment (green pixels) and urban built-up area (red pixels) in the PRD (**a**) and YRD (**b**), respectively. The base map shows the elevation from Shuttle Radar Topography Mission (STRM) data with 90 m resolution (https://www2.jpl.nasa.gov). PRD mainly includes five cities: Shenzhen (SZ), Dongguan (DG), Guangzhou (GZ), Foshan (FS), and Zhongshan (ZS). YRD mainly includes four cities: Shanghai (SH), Suzhou (SuZ), Wuxi (WX), and Changzhou (CZ).

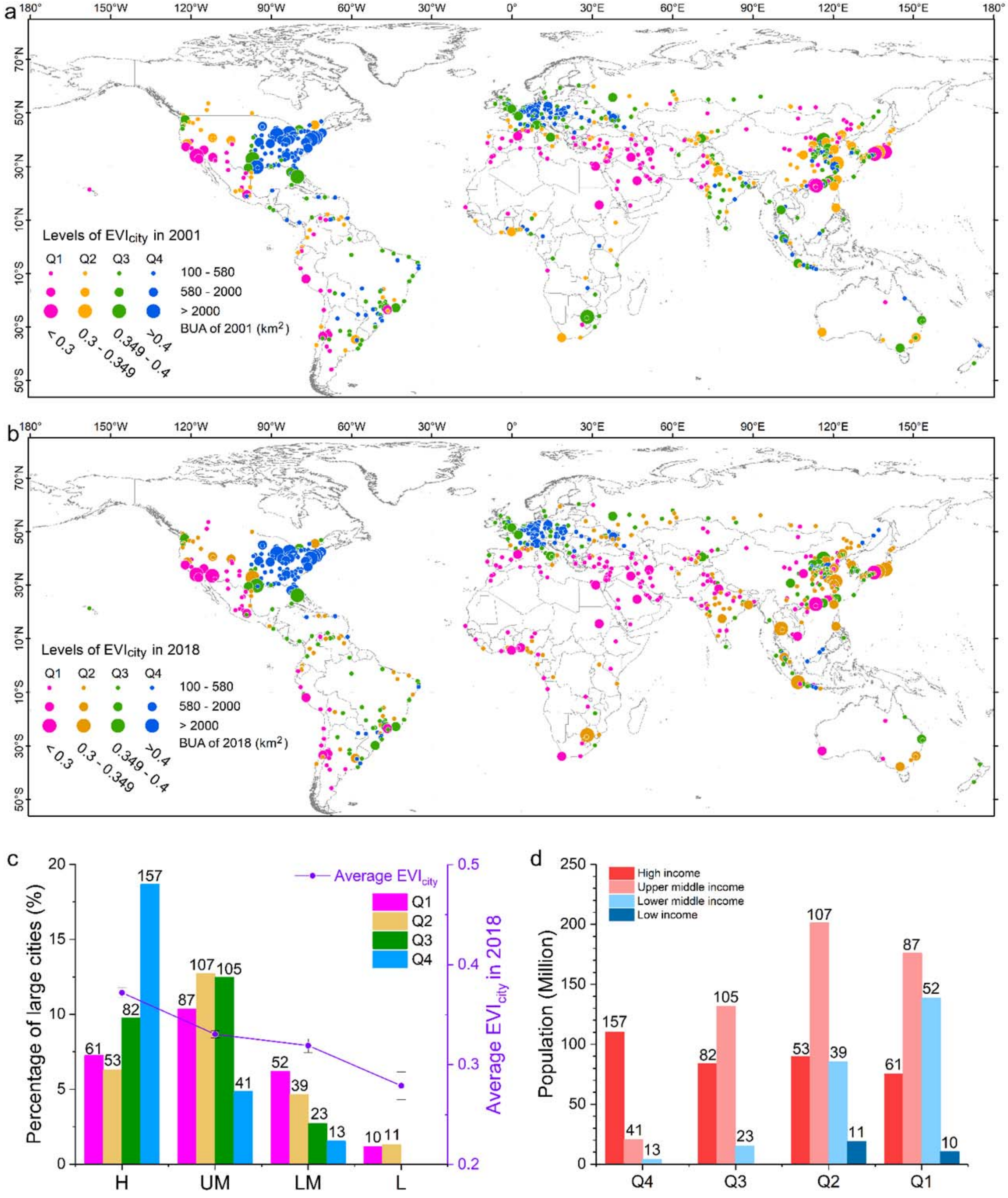

Supplementary Figure 5 | Distribution of large cities with average annual maximum greenness in 2001 and 2018. a. Distribution of 777 large cities with different levels of average EVI_{max} (EVI_{city}) in 2001. **b**. Distribution of the 841 large cities with different levels of the average EVI_{max} (EVI_{city}) in 2018. In (**b**), all the cities are sorted according to the values of EVI_{city} for each city and roughly divided into four quarters: Q1: $EVI_{city} \leqslant 0.3$, 210 magenta points; Q2: $0.3 < EVI_{city} \leqslant 0.349$, 210 orange points; Q3: $0.349 < EVI_{city} \leqslant 0.4$, 210 green points; Q4: $EVI_{city} > 0.4$, 211 blue points. **c**. Bars are the percentages of the numbers of the total 841 large cities in 2018 for 4 EVI_{city} levels and 4 economic levels. Purple lines are the average EVI_{city} in 2018 at 4 economic levels. Error bars show S.E.M. for each economic level. **d**. The total

population living in the urban areas in 2018 at 4 EVI_{city} levels with 4 economic levels. The number of cities in each class is labeled on the top of each bar.

Supplementary Table 1 | Ranking of the10 large cities by the largest greening built-up area (BUA) from 2001 to 2018

Rank	City name	Country	BUA (km^2)	Greening BUA (km^2)	$R_{greening}$
1	PRD*	China	6,219	2,398	0.38
2	Tokyo Bay**	Japan	6,281	1,209	0.19
3	YRD***	China	6,326	747	0.12
4	Miami	USA	2,954	618	0.21
5	Beijing	China	3,115	611	0.20
6	Chicago	USA	4,042	501	0.12
7	Seoul	Korea	1,477	459	0.31
8	Tianjin	China	1,837	425	0.23
9	Sao Paulo	Brazil	1,922	408	0.21
10	Osaka	Japan	2,244	396	0.18

* The Pearl River Delta (PRD) mainly includes five cities, Shenzhen (SZ), Dongguan (DG), Guangzhou (GZ), Foshan (FS), and Zhongshan (ZS).

** The Tokyo Bay Area mainly includes Tokyo, Kawasaki, Yokohama, Saitama, and Chiba City.

*** The Yangtze River Delta (YRD) mainly includes four cities, Shanghai (SH), Suzhou (SuZ), Wuxi (WX), and Changzhou (CZ).

Supplementary Table 2 | Ranking of the 20 large cities by the population living with greening BUA in 2015.

Rank	Name of large city	Country	Population living in greening BUA (Thousands)	Greening BUA (km^2)	$R_{greening}$	EVI_{city} in 2018
1	Pearl River Delta	China	17,479	2,398	0.38	0.30
2	Yangtze River Delta	China	12,436	747	0.12	0.32
3	Beijing	China	7,731	611	0.20	0.38
4	Tokyo	Japan	6,568	1,209	0.19	0.33
5	Seoul	Korea	5,954	459	0.31	0.33
6	Sao Paulo	Brazil	3,776	408	0.21	0.29
7	Tianjin	China	3,461	425	0.23	0.31
8	Chengdu	China	3,256	193	0.17	0.36
9	Nanjing	China	3,198	182	0.27	0.34
10	Xi'an	China	2,833	152	0.21	0.30
11	Shenyang	China	2,699	227	0.23	0.34
12	Osaka	Japan	2,530	396	0.18	0.28
13	Hankou	China	2,395	106	0.29	0.30
14	Taiyuan	China	2,160	334	0.37	0.35
15	Chongqing	China	2,146	163	0.29	0.34
16	Paris	France	2,143	276	0.19	0.37
17	Mumbai	India	1,957	69	0.15	0.32
18	Cairo	Egypt	1,880	216	0.20	0.19
19	Urumqi*	China	1,817	177	0.53	0.23
20	Taipei	China	1,689	183	0.28	0.36

*Urumqi (UQ), the capital of Xinjiang Autonomous Region of China, recorded the highest value of $R_{greening}$ (0.53) among the 20 large cities. Notably, the EVI_{city} of Urumqi is only 0.23, which is the lowest value among the 150 large cities in China in 2018.
