# Supplementary material for: Dramatic uneven urbanization of large cities throughout the world in recent decades
Source: Nat Commun. 2020 Oct 23;11:5366. doi: 10.1038/s41467-020-19158-1 (PMC7584620; doi:10.1038/s41467-020-19158-1)
Supplement: Supplementary file 3 — Reporting Summary [file 41467_2020_19158_MOESM3_ESM.pdf]

nature research

Corresponding author(s): Ji Chen, Qinglan Li

Last updated by author(s): August 26, 2020

# Reporting Summary

Nature Research wishes to improve the reproducibility of the work that we publish. This form provides structure for consistency and transparency in reporting. For further information on Nature Research policies, see Authors & Referees and the Editorial Policy Checklist .

## Statistics

For all statistical analyses, confirm that the following items are present in the figure legend, table legend, main text, or Methods section.

| n/a | Confirmed | |
|---|---|---|
| ☐ | ☒ | The exact sample size ($n$) for each experimental group/condition, given as a discrete number and unit of measurement |
| ☐ | ☒ | A statement on whether measurements were taken from distinct samples or whether the same sample was measured repeatedly |
| ☐ | ☒ | The statistical test(s) used AND whether they are one- or two-sided<br>*Only common tests should be described solely by name; describe more complex techniques in the Methods section.* |
| ☐ | ☒ | A description of all covariates tested |
| ☐ | ☒ | A description of any assumptions or corrections, such as tests of normality and adjustment for multiple comparisons |
| ☐ | ☒ | A full description of the statistical parameters including central tendency (e.g. means) or other basic estimates (e.g. regression coefficient) AND variation (e.g. standard deviation) or associated estimates of uncertainty (e.g. confidence intervals) |
| ☐ | ☒ | For null hypothesis testing, the test statistic (e.g. $F$, $t$, $r$) with confidence intervals, effect sizes, degrees of freedom and $P$ value noted<br>*Give P values as exact values whenever suitable.* |
| ☐ | ☒ | For Bayesian analysis, information on the choice of priors and Markov chain Monte Carlo settings |
| ☐ | ☒ | For hierarchical and complex designs, identification of the appropriate level for tests and full reporting of outcomes |
| ☐ | ☒ | Estimates of effect sizes (e.g. Cohen's $d$, Pearson's $r$), indicating how they were calculated |

*Our web collection on statistics for biologists contains articles on many of the points above.*

## Software and code

Policy information about availability of computer code

| | |
|---|---|
| Data collection | All methods of data collection in this study are described in the paper. |
| Data analysis | All methods of data analysis in this study are described in the paper. |

For manuscripts utilizing custom algorithms or software that are central to the research but not yet described in published literature, software must be made available to editors/reviewers. We strongly encourage code deposition in a community repository (e.g. GitHub). See the Nature Research guidelines for submitting code & software for further information.

## Data

Policy information about availability of data

All manuscripts must include a data availability statement. This statement should provide the following information, where applicable:

- Accession codes, unique identifiers, or web links for publicly available datasets
- A list of figures that have associated raw data
- A description of any restrictions on data availability

The land cover type data is the collection 6.0 MODIS yearly product known as MCD12Q1; The EVI data is from the MOD13A1; The country population data were obtained from World Urbanization Prospects: The 2018 Revision (https://population.un.org/wup/). The 1 km resolution Gridded Population of the World (GPW) collection (fourth version; GPWv4), which is adjusted to the national-level population counts estimated by the United Nations (UN), was downloaded from the Socioeconomic Data and Applications Center (https://sedac.ciesin.columbia.edu/data/collection/gpw-v4/united-nations-adjusted). All of the data used in this publication could be downloaded freely from internet.

# Field-specific reporting

Please select the one below that is the best fit for your research. If you are not sure, read the appropriate sections before making your selection.

☐ Life sciences ☐ Behavioural & social sciences ☒ Ecological, evolutionary & environmental sciences

For a reference copy of the document with all sections, see nature.com/documents/nr-reporting-summary-flat.pdf

# Ecological, evolutionary & environmental sciences study design

All studies must disclose on these points even when the disclosure is negative.

| | |
|---|---|
| Study description | Analysis of uneven distribution of global urbanization in terms of urban expansion, population growth, built-up area greening and its beneficiaries on different economic levels. |
| Research sample | The vegetation index (VI) datasets from the MOD13A1 version 6 product were used in this study to match the spatial resolution of the land cover data (500 m). The Gridded Population of the World is 1-km resolution. |
| Sampling strategy | Majority |
| Data collection | Open source data |
| Timing and spatial scale | Collecting in 2020 by websites |
| Data exclusions | No data were excluded the analyses |
| Reproducibility | The experiments are reproducible. |
| Randomization | The uncertainties of the results are described in the paper. |
| Blinding | None |

Did the study involve field work? ☐ Yes ☒ No

# Reporting for specific materials, systems and methods

We require information from authors about some types of materials, experimental systems and methods used in many studies. Here, indicate whether each material, system or method listed is relevant to your study. If you are not sure if a list item applies to your research, read the appropriate section before selecting a response.

## Materials & experimental systems

| n/a | Involved in the study |
|---|---|
| ☒ | ☐ Antibodies |
| ☒ | ☐ Eukaryotic cell lines |
| ☒ | ☐ Palaeontology |
| ☒ | ☐ Animals and other organisms |
| ☒ | ☐ Human research participants |
| ☒ | ☐ Clinical data |

## Methods

| n/a | Involved in the study |
|---|---|
| ☒ | ☐ ChIP-seq |
| ☒ | ☐ Flow cytometry |
| ☒ | ☐ MRI-based neuroimaging |
